# Effects of aromatherapy on objective physiological outcomes in adult ICU patients: a systematic review

**DOI:** 10.3389/fmed.2026.1807990

**Published:** 2026-06-03

**Authors:** Pedro Almeida Moyano, Zainab Salam Sheikh, Fatima Binte Athar, Larissa Gouveia, Luciana Aparecida Campos, Ovidiu Constantin Baltatu

**Affiliations:** 1 Center of Innovation, Technology, and Education (CITE) at Anhembi Morumbi University, Ânima Institute, Sao Jose dos Campos Technology Park, Sao Jose dos Campos, Brazil; 2 Inspirali Research Organization (IRO), Inspirali, Ânima Educação, São Paulo, Brazil; 3 College of Medicine, Alfaisal University, Riyadh, Saudi Arabia

**Keywords:** aromatherapy, blood pressure, essential oils, heart rate, hemodynamic parameters, intensive care unit, physiological parameters, systematic review

## Abstract

**Background:**

Aromatherapy has been proposed as a non-pharmacological adjunctive intervention in critical care settings; however, evidence regarding its effects on objective outcome measures remains inconclusive. This systematic review aimed to evaluate the effectiveness of aromatherapy in modulating objective physiological and biochemical markers in adult intensive care unit (ICU) patients. We hypothesized that the controlled ICU environment would facilitate rigorous measurement of both physiological parameters and biochemical stress markers.

**Methods:**

A systematic search was conducted using a novel approach combining lexical database searching (PubMed) and AI-powered semantic search strategies (Elicit, Undermind). Randomized controlled trials examining aromatherapy effects on physiological parameters in critically ill and cardiac patients were included. Outcomes were categorized as cardiovascular, respiratory, and non-cardiovascular/non-respiratory parameters. Subgroup analyses were performed by essential oil type.

**Results:**

Eighteen studies comprising 1236 participants were included. Aromatherapy demonstrated moderate effects on cardiovascular parameters (systolic blood pressure: 50% success rate, 5–22 mmHg reductions; diastolic blood pressure: 50%, 3.7–14 mmHg; heart rate: 44%, up to 20 bpm) but limited effects on respiratory parameters (respiratory rate: 25%; peripheral oxygen saturation: 12.5%), with the exception of eucalyptus oil, which showed 75% success for respiratory outcomes in mechanically ventilated patients. Non-cardiovascular/non-respiratory outcomes demonstrated the highest efficacy: anxiety (86%), sleep quality (100%), pain (100%), and sedation outcomes (100%). Subgroup analysis revealed essential oil blends achieved superior cardiovascular and psychological outcomes (100%) compared to lavender alone (64% cardiovascular; 71% anxiety/sedation). Contrary to our hypothesis, no studies measured biochemical stress markers such as cortisol or catecholamines, representing a significant gap in the evidence base. A notable finding was the disparity between evidence certainty: high for anxiety reduction versus low/very low for physiological outcomes, highlighting inadequate control for ICU-specific confounders in existing trials.

**Conclusion:**

Aromatherapy may serve as a safe adjunctive intervention in critically ill patients, with potential cardiovascular benefits requiring cautious interpretation and strong effects on anxiety and sleep outcomes. The absence of biochemical outcome measurements and inadequate control for ICU-specific confounders limits mechanistic understanding and causal inference.

## Introduction

Aromatherapy, the therapeutic application of plant-derived essential oils, has become increasingly popular as a complementary intervention in modern healthcare. Essential oils, complex mixtures of volatile organic compounds such as terpenes and phenylpropanoids, are extracted from aromatic plants and are believed to exert diverse physiological and psychological effects (1). Clinical applications of aromatherapy range from stress reduction and mood enhancement to the management of pain, anxiety, and sleep disturbances (1).

The mechanisms underlying aromatherapy’s effects are multifaceted. Inhaled volatile molecules interact with olfactory receptors in the nasal epithelium, transmitting signals to the olfactory bulb and subsequently to the limbic system—a brain region integral to emotion, memory, and autonomic regulation (2). This neural circuitry provides a plausible basis for the modulation of mood, stress responses, and physiological arousal observed with certain essential oils. Additionally, some essential oil constituents have demonstrated direct pharmacological actions, including modulation of neurotransmitter systems (notably GABAergic activity), anti-inflammatory effects, and antioxidant properties (3).

Despite its widespread use, the scientific evidence supporting aromatherapy’s efficacy remains limited by methodological shortcomings, most notably, a reliance on subjective outcome measures such as patient satisfaction, psychometric scales, and self-reported assessments (4). While these endpoints provide valuable insights into patient experience, they are susceptible to bias, placebo effects, and poor reproducibility, which undermines the scientific rigor and generalizability of findings (5). Recent systematic reviews and bibliometric analyses have consistently identified this overreliance on subjective measures as a fundamental barrier to establishing the true clinical efficacy of aromatherapy (6, 7).

There is now a growing consensus that future aromatherapy research must prioritize quantitative, objective measurements to establish efficacy and elucidate mechanisms of action (3). Systematic reviews and methodological critiques published in recent years explicitly recommend the adoption of standardized physiological parameters (e.g., heart rate, blood pressure, respiratory rate) and biochemical markers (e.g., cortisol, inflammatory cytokines) as primary endpoints (6, 8, 9). These objective measures are essential for enhancing reproducibility, enabling meaningful comparisons across studies, and moving beyond anecdotal or placebo-driven effects (7).

The intensive care unit (ICU) offers a uniquely advantageous environment for rigorous aromatherapy research focused on quantitative outcomes. ICU patients experience significant psychological and physiological stress due to factors such as invasive procedures, mechanical ventilation, environmental stressors, and social isolation (10). The prevalence of moderate to severe stress, anxiety, and depression among ICU patients is substantial, with these symptoms closely linked to poor sleep quality, prolonged recovery, and adverse clinical outcomes (10, 11).

Traditional management of ICU-related stress has relied predominantly on pharmacological interventions, such as sedatives and analgesics, which are associated with significant adverse effects including delirium, respiratory depression, and prolonged ICU stays. Recent clinical guidelines emphasize the urgent need for effective non-pharmacological interventions to reduce psychological distress and improve patient outcomes in the ICU (12).

From a methodological perspective, the ICU represents an optimal environment for evaluating interventions using objective outcome measures. Continuous, high-fidelity monitoring systems enable real-time acquisition of vital signs (heart rate, blood pressure, respiratory parameters) and advanced hemodynamic variables with high temporal resolution (13, 14). Routine arterial and venous access facilitates serial measurement of biochemical stress markers, including cortisol, catecholamines, and inflammatory cytokines (15). Critically, the standardized care protocols, controlled environmental conditions, and continuous patient observation inherent to ICU practice minimize confounding variables that complicate outcome interpretation in less controlled clinical settings. These methodological advantages position the ICU as an ideal environment for detecting subtle but clinically meaningful physiological and biochemical responses to aromatherapy interventions.

To comprehensively characterize the evidence landscape and contextualize our physiological findings, we also extracted psychological outcomes (anxiety, sleep quality, pain) reported in included studies. These outcomes, while often described as ‘subjective’ experiences, were measured using validated instruments with numerical scales (e.g., Visual Analog Scale, State–Trait Anxiety Inventory, Pittsburgh Sleep Quality Index), generating quantitative data. We note that individual studies varied in their outcome prioritization, with some designating psychological measures as primary endpoints; our extraction of these data serves to accurately represent the included studies and to highlight the disparity between evidence certainty for psychological versus physiological outcomes—a key finding of this review.

Given (1) the established need for objective evidence in aromatherapy research, (2) the unique methodological advantages of the ICU for precise physiological monitoring under controlled conditions, and (3) the clinical imperative to develop effective non-pharmacological interventions for critically ill patients, there is a compelling rationale for systematically evaluating the effectiveness of aromatherapy in modulating objective physiological and biochemical markers in adult ICU patients.

This systematic review aimed to address the primary research question: What is the effectiveness of aromatherapy in modulating objective physiological and biochemical markers in adult ICU patients? By focusing exclusively on objective, measurable outcomes, this review seeks to provide a rigorous evaluation of aromatherapy’s clinical significance in the intensive care setting. We hypothesized that the controlled ICU environment would facilitate measurement of both physiological parameters and biochemical stress markers; however, the extent to which existing studies have utilized the ICU’s capacity for biochemical assessment was unknown.

## Methods

This systematic review was conducted according to PRISMA (Preferred Reporting Items for Systematic Reviews and Meta-Analyses) 2020 guidelines (16). A protocol was developed *a priori*; however, it was not prospectively registered in PROSPERO (Prospective Register of Systematic Reviews). To mitigate the potential for selective reporting bias, the a priori protocol was followed strictly throughout the review: no post-hoc modifications were made to eligibility criteria, outcome definitions, or analytical approaches once data extraction commenced. All outcomes specified in the original protocol are reported. The absence of prospective registration is acknowledged as a methodological limitation.

### Information sources and search strategy

The literature search employed two methodologically distinct approaches: lexical searching and AI-powered semantic searching. Lexical searching utilizes Boolean operators, controlled vocabulary [Medical Subject Headings (MeSH) terms], and field-specific tags to retrieve documents based on exact term matching within indexed fields. This approach requires predefined keyword combinations and relies on standardized terminology for document retrieval. AI-powered semantic searching employs natural language processing and large language models (LLMs) to interpret research questions and assess document relevance based on conceptual alignment rather than keyword concordance.

Three platforms were searched: PubMed (MEDLINE, non-MEDLINE, preprints) for lexical searching, and Elicit (Semantic Scholar, PubMed, OpenAlex, preprints) and Undermind (Semantic Scholar) for AI-powered semantic searching. Elicit processes queries through a unified index spanning multiple databases and applies machine-learning algorithms for relevance filtering and automated data extraction (17). Undermind employs an iterative agent-based strategy incorporating citation tracing, and uses a large language model to assess candidate relevance, generating a topic-match score that quantifies alignment with the research question (18). Semantic Scholar and OpenAlex aggregate metadata from PubMed, Crossref, and indexed publisher repositories, providing coverage of biomedical and allied health literature. This AI-assisted approach was adopted to broaden retrieval beyond keyword-based searching and to identify eligible studies that may also be indexed in CINAHL, EMBASE, or Scopus. We acknowledge, however, that Semantic Scholar and OpenAlex do not replicate the controlled vocabulary indexing or specialty journal coverage of CINAHL, EMBASE, and Scopus—particularly for nursing dissertations and conference proceedings—and the absence of these databases is noted as a methodological limitation.

The PubMed search strategy was: ((“aromatherapy”[MeSH Terms] OR “aromatherapy”[Title/Abstract] OR “essential oils”[Title/Abstract] OR “aromatherapy”[Supplementary Concept] OR “essential oil therapy”[Title/Abstract] OR “fragrant inhalation”[Title/Abstract]) AND (“intensive care units”[MeSH Terms] OR “critical care”[MeSH Terms] OR “ICU”[Title/Abstract] OR “intensive care”[Title/Abstract] OR “critical care unit”[Title/Abstract] OR “intensive therapy unit”[Title/Abstract])).

Elicit and Undermind were queried using the natural language research question: “What is the effectiveness of aromatherapy in modulating quantitative physiological and biochemical markers in adult ICU patients?” Reference lists of included studies were hand-searched for additional eligible studies.

### Eligibility criteria (PICOS framework)

Eligibility criteria were defined using the PICOS (Population, Intervention, Comparison, Outcome, Study design) framework (Table 1).

**Table 1:** Eligibility criteria for study selection based on the PICOS framework.

Component	Inclusion criteria	Exclusion criteria
Population	Adult patients (≥18 years) admitted to the ICU for any medical or surgical condition.	Pediatric patients, non-ICU settings, animal studies, or studies without ICU context.
Intervention	Aromatherapy using essential oils (inhalation, topical application, diffusion). Single oils or combinations were eligible.	Studies combining aromatherapy with other interventions where effects could not be isolated, using synthetic fragrances or non-aromatherapy interventions.
Comparison	Standard care, placebo (e.g., carrier oil without essential oil), sham intervention, or no intervention.	Studies without a comparison group.
Outcomes	Studies must have measured at least one quantitative physiological or biochemical outcome.	Studies reporting only subjective outcomes without physiological measures.
Study design	Randomized controlled trials (RCTs) and quasi-experimental studies (non-randomized controlled trials, before-after studies with control groups).	Reviews, meta-analyses, protocols, editorials, commentaries, case reports, case series.

### Outcomes of interest

This systematic review focused on physiological parameters (cardiovascular: systolic and diastolic blood pressure, heart rate; respiratory: respiratory rate, oxygen saturation) and biochemical stress markers (cortisol, catecholamines, inflammatory cytokines) as outcomes of primary interest, reflecting our aim to evaluate objective, measurable effects of aromatherapy in the controlled ICU environment. Psychological and clinical outcomes (anxiety, sleep quality, pain) reported in included studies were also extracted to provide comprehensive characterization of the evidence landscape and contextualize physiological findings. We note that individual studies varied in their own outcome prioritization, with some designating psychological measures as primary endpoints. Our inclusion of these data serves to accurately represent the included studies and to highlight the disparity between evidence certainty for psychological versus physiological outcomes.

### Study selection

Search results were imported into Rayyan for screening. Three reviewers independently screened titles/abstracts, then full texts. Disagreements were resolved through discussion or third-reviewer consultation.

Studies were eligible if they measured at least one quantitative physiological or biochemical outcome. We defined physiological markers as vital signs and hemodynamic parameters, and biochemical markers as laboratory-measured biomarkers including but not limited to cortisol, catecholamines, and inflammatory cytokines.

### Data extraction

A standardized data extraction form was developed to capture essential information from the selected studies. This form included fields for study characteristics such as author, year, country, and design; population demographics; detailed intervention information including essential oil type, concentration, and administration method; control or comparison conditions; and physiological outcome measures.

The data extraction process was enhanced by leveraging the capabilities of both AI tools. Elicit’s automated data extraction for methodological details provided a baseline of information, which was then supplemented by Undermind’s metadata extraction capabilities. This AI-assisted extraction was followed by manual verification by three independent reviewers, ensuring accuracy and completeness of the extracted data. The process also involved cross-referencing of overlapping articles to ensure data consistency across different sources.

### Risk of bias assessment

Three independent reviewers assessed risk of bias in included studies using appropriate tools based on study design. For RCTs: Cochrane Risk of Bias tool version 2 (RoB 2) (19). Domains assessed: bias arising from the randomization process, bias due to deviations from intended interventions, bias due to missing outcome data, bias in measurement of the outcome, bias in selection of the reported result. Each domain was rated as “low risk,” “some concerns,” or “high risk” based on signaling questions and algorithms. An overall risk of bias judgment was derived for each outcome, with studies classified as low risk only if all domains were rated as low risk. For quasi-experimental studies: Risk of Bias in Non-randomized Studies of Interventions (ROBINS-I) tool (20). Domains assessed: bias due to confounding, bias in selection of participants, bias in classification of interventions, bias due to deviations from intended interventions, bias due to missing data, bias in measurement of outcomes, bias in selection of the reported result.

### Certainty of evidence assessment

The certainty of evidence for each outcome was assessed using the Grading of Recommendations Assessment, Development and Evaluation (GRADE) approach (21). GRADE assessment was applied to evaluate evidence certainty across outcomes regardless of the number of contributing studies, as recommended by Cochrane methodology. The GRADE framework explicitly addresses limited evidence through the imprecision domain, with evidence downgraded when optimal information size is not met or confidence intervals are wide. This approach ensures transparent communication of evidence limitations while providing clinicians with actionable certainty ratings. Evidence from RCTs started as high certainty and was downgraded based on five criteria: risk of bias: serious (−1) or very serious (−2) limitations, inconsistency: serious (−1) or very serious (−2) unexplained heterogeneity, indirectness: serious (−1) or very serious (−2) differences in population, intervention, or outcomes, imprecision: serious (−1) or very serious (−2) based on sample size, confidence intervals, or optimal information size, publication bias: suspected (−1) based on funnel plot asymmetry or other evidence. Evidence was rated as high (⊕ ⊕ ⊕⊕), moderate (⊕ ⊕ ⊕○), low (⊕ ⊕ ○○), or very low (⊕○○○) certainty. GRADE assessments were conducted independently by two reviewers with disagreements resolved through discussion. Summary of Findings tables were created for primary outcomes using GRADEpro Guideline Development Tool (GDT) software software.

### Data synthesis and analysis

Due to substantial heterogeneity in interventions (essential oil types, concentrations, delivery methods, dosing regimens), comparators, outcome measures, and measurement timing, meta-analysis was deemed inappropriate. Results were synthesized narratively and organized by outcome category.

## Results

### Database search and article selection

**Figure 1:**
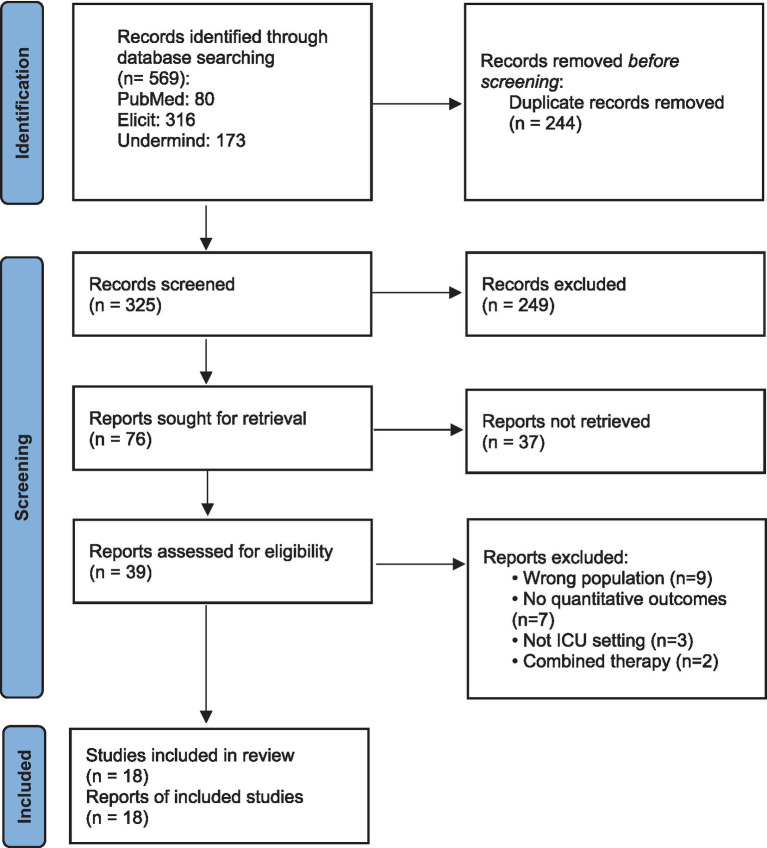
PRISMA flow diagram of the study selection process for the systematic review on the effectiveness of aromatherapy interventions in adult ICU patients.

The literature search was conducted across three platforms: PubMed (MeSH-based indexing, ATM, Boolean logic, field tags), Elicit (AI-powered semantic search, LLMs, automated extraction) and Undermind (Hybrid keyword/semantic, LLM relevance, agent-based). All search results were analyzed through Rayyan, a systematic review management tool. After removing duplicates within each platform, Elicit yielded 316 unique articles, Undermind yielded 173 articles, and PubMed yielded 80 articles. Deduplication was performed using digital object identifier (DOI) matching as the primary method, with title-based matching applied for articles lacking DOIs. This process removed 94 duplicate entries from Elicit and 1 duplicate each from Undermind and PubMed. The PRISMA flow diagram (Figure 1) illustrates the study selection process. From the initial 569 unique articles identified across all platforms, all were screened for eligibility based on our inclusion criteria.

### Characteristics of included studies

This systematic review included 18 studies examining aromatherapy interventions in adult intensive care unit patients across diverse clinical populations and settings. The studies encompassed diverse ICU populations: two focused on mechanically ventilated patients, one on acute coronary syndrome (ACS) in coronary care units (CCU), one on myocardial infarction (MI) in CCU, one on unspecified CCU patients, three on post-coronary artery bypass graft (CABG) patients, one on percutaneous coronary intervention (PCI) in cardiovascular ICU, two on open heart surgery ICU patients, one on post-cardiac surgery ICU patients, two on general ICU patients, one on internal medicine ICU patients, one on burn patients, one on CABG candidates, and one on unspecified ACS patients (Table 2).

**Table 2:** Characteristics of included studies.

Study	Study design	Study population	Intervention protocol	Physiological markers measured
Cho et al., 2013 (22)	Quasi-experimental	PCI in cardiovascular ICU (*n* = 56)	Lavender/chamomile/neroli inhalation, 2 drops, 10 times pre/post PCI	Anxiety, sleep, SBP, DBP
Nategh et al., 2015 (31)	Placebo-controlled RCT	ACS in CCU (*n* = 110; 52.7% women)	Lavender inhalation, 2 drops, 20 min at bedtime for 3 days	SBP, DBP, HR
Bikmoradi et al., 2015 (30)	Single-blind RCT	Post-CABG (*n* = 60)	Lavender inhalation, 2% in alcohol, 2 drops, 20 min, 2 days	SBP, DBP, HR, RR, temperature
Rajai et al., 2016 (29)	RCT	CABG candidates (*n* = 60)	Lavender inhalation, 2 drops, 20 min, pre-surgery	HR, SBP, DBP, RR
Mirbastegan Bavarsad et al., 2016 (49)	Single-blind RCT	MI in CCU (*n* = 60; 30/30 male/female)	Lavender inhalation, 3 drops, 20–30 min, 3 times/day for 3 days	SBP, DBP, HR, RR, temperature
Ghasemi et al., 2017 (50)	RCT	Open heart surgery ICU (*n* = 160)	Rose/lavender inhalation, 3 drops, attached to endotracheal tube	HR, RR, SBP, DBP, SaO_2_
Aflaki et al., 2017 (23)	Quasi-experimental	Mechanically ventilated ICU (*n* = 40)	Lavender inhalation, 2% in water, 2 drops/200 mL, 3 h	HR, RR, SBP, DBP, temperature, SaO_2_, agitation
Salamati et al., 2017 (24)	Single-blind	Open-heart surgery ICU (*n* = 40)	Lavender inhalation, 2 drops 2%, 10 min, post-extubation	SBP, DBP, HR, RR, SaO_2_, CVP
Cho et al., 2017 (25)	Nonrandomized controlled	General ICU (*n* = 60)	Lavender inhalation, 3 drops, aroma stone, 1 h after admission, 8 p.m.	Perceived/objective stress, SBP, DBP, HR, sleep
Ebrahimi et al., 2018 (51)	Double-blind RCT	Post-CABG (*n* = 98; mean age ~59)	Lavender inhalation, 5 drops 20%, 30 min every 24 h for 3 days	SBP, DBP, PR, RR
Zare et al., 2020 (28)	RCT	General ICU (*n* = 60)	Rose inhalation, 3 drops 10%, 20 min, 3 times/day for 1 day	SBP, DBP, HR, RR, SaO_2_
Davari et al., 2021 (32)	RCT	Post-CABG (*n* = 50)	Lavender inhalation, 2 drops, 10 h, 3 nights	SBP, RR, SaO_2_, HR, temperature
Buyukbayram et al., 2021 (52)	Nonrandomized controlled	Internal medicine ICU (*n* = 100)	Lavender inhalation/topical, 5 drops, 15 min, single session	SBP, DBP, HR, RR, SaO_2_
Amini et al., 2022 (27)	Randomized controlled trial (RCT), single-blind	Mechanically ventilated intensive care unit (ICU) patients (trauma, post-operative, medical; 18–65 years; 70 patients)	Nebulized eucalyptus, 4 mL 5% solution plus 6 mL saline, 20–30 min every 8 h for 3 days	Arterial blood gas (pH, PaCO_2_, HCO_3_^−^, SaO_2_, PaO_2_), GCS, PIP, TV, vital signs
Froutan et al., 2022 (26)	RCT	Burn patients (*n* = 62)	Damask rose 40%/lavender 10% inhalation, 20 min pre-dressing	BIS, sedative/analgesic use, HR, BP, RR, SaO_2_
Pourshaikhian et al., 2024 (53)	Double-blind RCT	ACS (*n* = 154)	Chamomile inhalation, 7 drops 10%, 2 nights, 10 breaths, worn overnight	SBP, DBP, HR
De Melo et al., 2024 (33)	RCT	Post-cardiac surgery ICU (*n* = 52)	Lavender inhalation, 1 drop, 30 min, single session	Pain, MAP, HR, RR, SaO_2_, temperature
Mahmoodabadipoor et al., 2024 (54)	Double-blind RCT	CCU patients (*n* = 76; mean age ~57)	Chamomile plus *citrus aurantium* inhalation, blend 2:0.5, 3 nights	HR, SBP

ACS, acute coronary syndrome; HCO_3_^−^, bicarbonate; BIS, bispectral index; CABG, coronary artery bypass graft; CCU, coronary care unit; CVP, central venous pressure; DBP, diastolic blood pressure; GCS, Glasgow Coma Scale; HR, heart rate; ICU, intensive care unit; MAP, mean arterial pressure; MI, myocardial infarction; PaCO_2_, partial pressure of carbon dioxide; PaO_2_, partial pressure of oxygen; PCI, percutaneous coronary intervention; PIP, peak inspiratory pressure; PR, pulse rate; RCT, randomized controlled trial; RR, respiratory rate; SaO_2_, arterial oxygen saturation; SBP, systolic blood pressure; SpO_2_, peripheral oxygen saturation; TV, tidal volume. Study Design Classifications: Single-blind, participants blinded to treatment allocation; Double-blind, both participants and investigators blinded to treatment allocation; Quasi-experimental, controlled study without randomization; Nonrandomized controlled, controlled study with non-random treatment assignment. Intervention Protocols: Essential oil concentrations are expressed as percentages where specified. Session durations and frequencies varied across studies as indicated in individual protocol descriptions.

### Aromatherapy effects on cardiovascular parameters in the ICU

Table 3 presents the effects of aromatherapy interventions on cardiovascular parameters as reported in studies involving critically ill and cardiac patient populations. The effects of the intervention on systolic blood pressure (SBP) varied considerably across the included studies, with baseline SBP values ranging from approximately 110 to 147 mmHg. Treatment effects ranged from no statistically significant change to reductions of approximately 20–22 mmHg in studies reporting significant findings. The most pronounced and statistically significant effects were observed in the Cho, Mashouf, and Salamati studies (reductions of 16–22 mmHg), while a substantial proportion of studies found no significant between-group differences (22–25). This variability may reflect differences in patient populations, intervention protocols, measurement timing, and baseline hemodynamic status. Even modest reductions in SBP (e.g., 5 mmHg) may be clinically relevant, as large-scale meta-analyses have demonstrated that a 5 mmHg reduction in SBP reduces the risk of major cardiovascular events by approximately 10%.

**Table 3:** Effects of aromatherapy interventions on cardiovascular parameters across included studies in critically ill and cardiac patients.

Outcome category	First author, year	Essential oil	*N*	Measurement tool	Baseline value (mean±SD)	Post-intervention value (mean±SD)	*p*-value	Significant effect
SBP	Cho, 2013/2017 (22, 25)	Lavender + Chamomile + Neroli	56	Non-invasive BP monitor	Exp: 134.60 ± 30.15 mmHg; Control: 125.13 ± 24.03 mmHg	Exp: 112.53 ± 14.79 mmHg; Control: 121.60 ± 17.10 mmHg	*p* = 0.017 (group); *p* < 0.001 (Group×Time)	Yes
	Mashouf, 2017 (23)	Lavender	40	Non-invasive BP monitor	\ ~ 147 mmHg	\ ~ 127 mmHg	*p* < 0.001	Yes
	Salamati, 2017 (24)	Lavender	40	Non-invasive BP monitor	123.7 mmHg	107.3 mmHg	*p* < 0.001	Yes
	Pourshaikhian, 2024 (53)	Chamomile	154	Non-invasive BP monitor	\ ~ 126 mmHg	\ ~ 115 mmHg	*p* < 0.05	Yes
	Mirbastegan, 2016 (49)	Lavender	60	Non-invasive BP monitor	131.46 mmHg	126.6 mmHg	*p* = 0.042	Yes
	Ebrahimi Hosein Abadi, 2018 (51)	Lavender	98	Non-invasive BP monitor	Not specified	Significant ↓ Day 2	*p* = 0.046 (Day 2); *p* = 0.002 (regression)	Yes\*
	Bikmoradi, 2015 (30)	Lavender	60	Non-invasive BP monitor	\ ~ 130–135 mmHg	↓ at 5, 30 min Day 3	MD = 13.00 (95% CI 2.10–23.90) at 5 min; MD = 14.50 (95% CI 2.20–26.80) at 30 min	Yes\*
	Ghasemi, 2017 (50)	Rose/Lavender	40	Non-invasive BP monitor	\ ~ 125–130 mmHg	↓ at 15, 45, 60 min	Significant at specific times; *p* = 0.30 overall	Yes\*
	Rajai, 2016 (29)	Lavender	60	Non-invasive BP monitor	Exp: 127.50 ± 15.07 mmHg; Control: 124.73 ± 12.45 mmHg	Exp: 126.96 ± 12.14 mmHg; Control: 127.20 ± 10.64 mmHg	*p* = 0.96	No
	Nategh, 2015 (31)	Lavender	110	Non-invasive BP monitor	\ ~ 131–139 mmHg	\ ~ 131–139 mmHg	*p* = 0.586	No
	Amini, 2022 (27)	Eucalyptus	70	Non-invasive BP monitor	Exp: 123.89 ± 18.91 mmHg; Control: 129.09 ± 18.70 mmHg	Exp: 124.94 ± 22.49 mmHg; Control: 126.29 ± 24.13 mmHg	*p* = 0.32	No
	Mahmoodabadipoor, 2024 (54)	Citrus + Chamomile	76	Non-invasive BP monitor	Not specified	No significant change	β = −1.09 (95% CI − 6.53 to 4.36); *p* = 0.693	No
	Buyukbayram, 2021 (52)	Lavender	100	Non-invasive BP monitor	Exp: \ ~ 120 mmHg; Control: \ ~ 118 mmHg	Exp: \ ~ 117 mmHg; Control: \ ~ 115 mmHg	NS	No
	Davari, 2021 (32)	Lavender	50	Non-invasive BP monitor	Exp: \ ~ 125 mmHg; Control: \ ~ 127 mmHg	Exp: \ ~ 123 mmHg; Control: \ ~ 124 mmHg	*p* > 0.05	No
	Froutan, 2022 (26)	Rose + Lavender	62	Non-invasive BP monitor	Similar between groups	No significant between-group difference	MD = 6.02 (95% CI − 1.16 to 13.19); *p* = 0.099 (with inhalation); MD = −1.42 (95% CI − 6.23 to 3.39); *p* = 0.557 (with drug)	No
	Zare, 2020 (28)	Rose	60	Non-invasive BP monitor	Exp: \ ~ 118 mmHg; Control: \ ~ 115 mmHg	↑3–6 mmHg then ↓; not clinically significant	NS	No
DBP	Cho, 2013/2017 (22, 25)	Lavender + Chamomile + Neroli	56	Non-invasive BP monitor	Exp: 82.00 ± 21.23 mmHg; Control: 74.67 ± 14.50 mmHg	Exp: 67.60 ± 9.04 mmHg; Control: 73.73 ± 11.09 mmHg	*p* = 0.004 (group); *p* = 0.046 (Group×Time, 2017); *p* < 0.001 (Group×Time, 2013)	Yes
	Mashouf, 2017 (23)	Lavender	40	Non-invasive BP monitor	\ ~ 88 mmHg	\ ~ 75 mmHg	*p* < 0.001	Yes
	Salamati, 2017 (24)	Lavender	40	Non-invasive BP monitor	73.43 mmHg	66.06 mmHg	*p* = 0.001	Yes
	Pourshaikhian, 2024 (53)	Chamomile	154	Non-invasive BP monitor	\ ~ 80 mmHg	\ ~ 72.73 mmHg	*p* < 0.001	Yes
	Mirbastegan, 2016 (49)	Lavender	60	Non-invasive BP monitor	76.90 ± 15.45 mmHg	73.20 ± 10.45 mmHg	*p* = 0.048	Yes
	Ebrahimi Hosein Abadi, 2018 (51)	Lavender	98	Non-invasive BP monitor	Not specified	Significant ↓ Day 1	*p* = 0.029 (Day 1); *p* = 0.001 (regression)	Yes\*
	Bikmoradi, 2015 (30)	Lavender	60	Non-invasive BP monitor	\ ~ 75–80 mmHg	↓ at 5 min Day 3 (7.10 mmHg difference)	MD = 7.10 (95% CI 1.10–13.10) at 5 min	Yes\*
	Ghasemi, 2017 (50)	Rose/Lavender	40	Non-invasive BP monitor	\ ~ 62–74 mmHg	\ ~ 62–74 mmHg	*p* = 0.94	No
	Rajai, 2016 (29)	Lavender	60	Non-invasive BP monitor	Exp: 76.13 ± 9.89 mmHg; Control: 76.03 ± 9.22 mmHg	Exp: 77.06 ± 7.87 mmHg; Control: 76.80 ± 7.81 mmHg	*p* = 0.91	No
	Nategh, 2015 (31)	Lavender	110	Non-invasive BP monitor	\ ~ 79–88 mmHg	\ ~ 79–88 mmHg	*p* = 0.557 (between); *p* = 0.012 (Group×Time)	No
	Amini, 2022 (27)	Eucalyptus	70	Non-invasive BP monitor	Exp: 77.40 ± 12.49 mmHg; Control: 82.43 ± 14.77 mmHg	Exp: 77.06 ± 15.86 mmHg; Control: 79.43 ± 16.67 mmHg	*p* = 0.42	No
	Buyukbayram, 2021 (52)	Lavender	100	Non-invasive BP monitor	Exp: \ ~ 70 mmHg; Control: \ ~ 72 mmHg	Exp: \ ~ 68 mmHg; Control: \ ~ 70 mmHg	*p* = 0.387	No
	Froutan, 2022 (26)	Rose + Lavender	62	Non-invasive BP monitor	\ ~ 70–79 mmHg	No significant between-group difference	MD = −0.27 (95% CI − 6.15 to 5.60); *p* = 0.926 (with inhalation); *p* = 0.832 (Group×Time)	No
	Zare, 2020 (28)	Rose	60	Non-invasive BP monitor	\ ~ 72–75 mmHg	↑4 mmHg then ↓6 mmHg; not clinically significant	*p* = 0.01, *p* = 0.003 (within-group)	No†
HR	Cho, 2013/2017 (22, 25)	Lavender + Chamomile + Neroli	56	Continuous ECG/pulse oximetry	Exp: 92.20 ± 19.52 bpm; Control: 78.93 ± 16.47 bpm	Exp: 71.97 ± 11.84 bpm; Control: 77.47 ± 12.55 bpm	*p* < 0.001; *p* = 0.015 (group, 2017); *p* < 0.001 (Group×Time, 2017)	Yes
	Mashouf, 2017 (23)	Lavender	40	Continuous monitoring	\ ~ 95–97 bpm	\ ~ 80–85 bpm	*p* < 0.001	Yes
	Froutan, 2022 (26)	Rose + Lavender	62	Continuous monitoring	\ ~ 114 bpm	\ ~ 104 bpm	MD = 8.35 (95% CI 3.04–13.66); *p* = 0.003 (with inhalation)	Yes†
	Salamati, 2017 (24)	Lavender	40	Continuous ECG	93.12 bpm	85.20 bpm	*p* = 0.03	Yes
	Rajai, 2016 (29)	Lavender	60	Continuous monitoring	Exp: 81.80 ± 11.37 bpm; Control: 82.83 ± 10.73 bpm	Exp: 78.83 ± 9.23 bpm; Control: 84.63 ± 10.41 bpm	*p* = 0.02	Yes
	Mahmoodabadipoor, 2024 (54)	Citrus + Chamomile	76	Continuous monitoring	Not specified	↓ in intervention group	β = −3.71 (95% CI − 7.23 to −0.19); *p* = 0.039	Yes
	Pourshaikhian, 2024 (53)	Chamomile	154	Continuous monitoring	\ ~ 80–81 bpm	\ ~ 78 bpm	*p* < 0.001	Yes
	Silva, 2024 (33)	Lavender	52	Continuous monitoring	Exp: 86.85 ± 15.75 bpm; Control: 85.27 ± 13.28 bpm	Exp: 89.19 ± 17.09 bpm; Control: 84.96 ± 13.38 bpm	*p* = 0.413	No
	Zare, 2020 (28)	Rose	60	Continuous monitoring	\ ~ 76–93 bpm	\ ~ 76–93 bpm	NS	No
	Buyukbayram, 2021 (52)	Lavender	100	Continuous monitoring	Exp: 92.54 bpm; Control: 92.36 bpm	Exp: 89.72 bpm; Control: 89.76 bpm	NS	No
	Davari, 2021 (32)	Lavender	50	Continuous monitoring	Exp: 93.88 bpm; Control: 94.84 bpm	Exp: 91.56 bpm; Control: 90.52 bpm	*p* > 0.05	No
	Ghasemi, 2017 (50)	Rose/Lavender	40	Continuous monitoring	\ ~ 92–104 bpm	\ ~ 92–104 bpm	*p* = 0.33	No
	Bikmoradi, 2015 (30)	Lavender	60	Continuous monitoring	\ ~ 77–85 bpm	\ ~ 77–85 bpm	All time points NS (95% CIs span 0; e.g., 3rd day 5 min: MD = 0.20, 95% CI − 8.68 to 9.08)	No
	Nategh, 2015 (31)	Lavender	110	Continuous monitoring	\ ~ 80–87 bpm	\ ~ 80–87 bpm	*p* = 0.846 (between); *p* = 0.012 (Group×Time)	No
	Amini, 2022 (27)	Eucalyptus	70	Continuous monitoring	Exp: 87.97 ± 18.89 bpm; Control: 93.31 ± 21.92 bpm	Exp: 88.77 ± 17.62 bpm; Control: 89.86 ± 18.05 bpm	*p* = 0.82	No
	Ebrahimi Hosein Abadi, 2018 (51)	Lavender	98	Continuous monitoring	\ ~ 81–93 bpm	\ ~ 81–93 bpm	NS	No
MAP	Silva, 2024 (33)	Lavender	52	Non-invasive BP monitor	Exp: 82.08 ± 14.51 mmHg; Control: 84.95 ± 12.81 mmHg	Exp: 76.58 ± 10.76 mmHg; Control: 83.31 ± 12.97 mmHg	*p* = 0.008	Yes

Effects of aromatherapy interventions on cardiovascular parameters across included studies in critically ill and cardiac patients. Effect sizes represent the magnitude of change from baseline to post-intervention or the difference between intervention and control groups, as reported in individual studies. Studies are grouped by outcome category and ordered by effect magnitude within each category. *p*-values and 95% confidence intervals are reported as published in the original studies; entries marked NS indicate the original study reported only “not significant” without an exact *p*-value. The frequent absence of 95% confidence intervals in the primary literature contributes to imprecision in the GRADE assessment.

Exp, experimental/intervention group; Control, control group; SBP, systolic blood pressure; DBP, diastolic blood pressure; HR, heart rate; MAP, mean arterial pressure; BP, blood pressure; ECG, electrocardiogram; bpm, beats per minute; mmHg, millimeters of mercury; CV, cardiovascular; N/A, not assessed; MD, mean difference (intervention − control); β, beta coefficient from generalized estimating equation (GEE) model; 95% CI, 95% confidence interval; NS, not significant (exact p-value not reported in original study); *Time-specific effects only (significant at select measurement points but not overall); †Within-group effect only; no significant between-group interaction observed.

A total of 14 studies examined diastolic blood pressure (DBP) as an outcome measure. The effects of the intervention on DBP varied considerably across studies, with baseline DBP values ranging from approximately 62 to 88 mmHg. Treatment effects ranged from no statistically significant change to reductions of approximately 13–14 mmHg in studies reporting significant findings. Approximately one-third of studies (5 of 14) demonstrated statistically significant reductions in DBP, with effect sizes ranging from 3.7 to 14 mmHg. The most pronounced effects were observed in the Cho and Mashouf studies (22, 23, 25). Two additional studies showed time-specific effects at discrete measurement points. Conversely, approximately 43% of studies (6 of 14) found no statistically significant difference between intervention and control groups for diastolic blood pressure. This heterogeneity in findings may reflect variability in patient populations, intervention protocols, timing of measurements, and baseline hemodynamic characteristics. Notably, while systolic blood pressure has historically received greater attention in cardiovascular risk assessment, recent evidence suggests that diastolic blood pressure contributes independently to cardiovascular risk prediction, particularly when considered alongside systolic values in calculations such as mean arterial pressure.

Sixteen studies examined the effect of aromatherapy on heart rate (HR) as a physiological outcome measure. The effects varied considerably across studies, with seven studies (43.8%) reporting statistically significant reductions in HR, while nine studies (56.2%) found no significant difference between intervention and control groups. Baseline HR values ranged widely across studies, from approximately 72 beats per minute (bpm) to 114 bpm, reflecting the heterogeneity of patient populations, which included post-cardiac surgery patients, mechanically ventilated patients, burn patients, and patients with acute coronary syndrome. The effects of aromatherapy on HR ranged from no significant change to reductions of approximately 20 bpm. When significant effects were observed, reductions typically ranged between 6–20 bpm. The most pronounced effects were observed in the Cho and Mashouf studies (22, 23, 25). The inconsistency in findings across studies may be attributed to differences in essential oil type and concentration, duration and frequency of intervention, patient populations, clinical settings, and methodological approaches.

### Aromatherapy effects on respiratory parameters in the ICU

A total of 12 studies examined respiratory rate (RR) as an outcome measure. The effects of the intervention on RR varied across studies, with baseline RR values ranging from approximately 10 to 27 breaths per minute (breaths/min), reflecting the heterogeneity of patient populations including post-cardiac surgery, mechanically ventilated, burn, and acute coronary syndrome patients. Treatment effects ranged from no statistically significant change to reductions of approximately 6–8 breaths/min in studies reporting significant findings. Only 25% of studies (3 of 12) reported statistically significant reductions in respiratory rate with aromatherapy intervention, with effect sizes ranging from approximately 1.8 to 8 breaths/min. The most pronounced effect was observed in the Mashouf (23) study involving mechanically ventilated patients. One additional study [Froutan et al. (26)] demonstrated within-group changes but failed to show significant between-group differences. The majority of studies (67%, 8 of 12) found no significant effect of aromatherapy on respiratory rate, suggesting that the intervention generally has minimal impact on this parameter in critically ill and cardiac patients (Table 4).

**Table 4:** Effects of aromatherapy interventions on respiratory parameters across included studies in critically ill and cardiac patients.

Outcome category	First author, year	Essential oil	*N*	Measurement tool	Baseline value (mean±SD)	Post-intervention value (mean±SD)	*P*-value	Significant effect
RR	Mashouf, 2017 (23)	Lavender	40	Continuous monitoring	\ ~ 26–27 breaths/min	\ ~ 18–20 breaths/min	*p* < 0.001	Yes
	Silva, 2024 (33)	Lavender	52	Continuous monitoring	Exp: 18.58 ± 3.42 breaths/min; Control: 17.96 ± 4.30 breaths/min	Exp: 16.65 ± 2.70 breaths/min; Control: 17.31 ± 3.95 breaths/min	*p* = 0.011	Yes
	Buyukbayram, 2021 (52)	Lavender	100	Continuous monitoring	Exp: 20.20 ± 3.60 breaths/min; Control: 18.56 ± 3.93 breaths/min	Exp: 18.40 ± 4.02 breaths/min; Control: 17.68 ± 3.76 breaths/min	*p* = 0.040 (within-group); *p* = 0.023, *p* = 0.016 (between-group at 30, 90 min)	Yes
	Froutan, 2022 (26)	Rose + Lavender	62	Continuous monitoring	Exp: 18.14 ± 2.27 breaths/min; Control: 17.60 ± 2.30 breaths/min	Exp: 14.94 ± 2.09 breaths/min; Control: 15.23 ± 2.16 breaths/min	*p* = 0.001 (within-group); MD = −0.52 (95% CI − 1.39 to 0.36); *p* = 0.243 (between-group); *p* = 0.512 (Group×measurement)	No†
	Salamati, 2017 (24)	Lavender	40	Continuous monitoring	20.48 breaths/min	20.32 breaths/min	*p* = 0.1	No
	Ghasemi, 2017 (50)	Rose/Lavender	40	Continuous monitoring	\ ~ 10–16 breaths/min	\ ~ 10–16 breaths/min	*p* = 0.11	No
	Rajai, 2016 (29)	Lavender	60	Continuous monitoring	Exp: 19.83 ± 2.80 breaths/min; Control: 19.30 ± 2.40 breaths/min	Exp: 19.83 ± 2.80 breaths/min; Control: 19.30 ± 2.40 breaths/min	NS	No
	Ebrahimi Hosein Abadi, 2018 (51)	Lavender	98	Continuous monitoring	Not specified	No significant change	NS	No
	Bikmoradi, 2015 (30)	Lavender	60	Continuous monitoring	\ ~ 20–26 breaths/min	\ ~ 20–26 breaths/min	All time points NS (95% CIs span 0; e.g., 3rd day 5 min: MD = 0.00, 95% CI − 3.47 to 3.47; 3rd day 30 min: MD = 0.10, 95% CI − 3.24 to 3.44)	No
	Davari, 2021 (32)	Lavender	50	Continuous monitoring	Exp: \ ~ 12.5 breaths/min; Control: \ ~ 14 breaths/min	Exp: \ ~ 13 breaths/min; Control: \ ~ 13.5 breaths/min	*p* = 0.709	No
	Zare, 2020 (28)	Rose	60	Continuous monitoring	\ ~ 18–20 breaths/min	\ ~ 18–20 breaths/min	NS	No
	Amini, 2022 (27)	Eucalyptus	70	Continuous monitoring	Exp: 17.60 ± 4.30 breaths/min; Control: 17.63 ± 3.79 breaths/min	Exp: 17.43 ± 3.83 breaths/min; Control: 18.26 ± 3.96 breaths/min	*p* = 0.88	No
SpO_2_	Mashouf, 2017 (23)	Lavender	40	Pulse oximetry	\ ~ 95.5%	\ ~ 97%	*p* < 0.001	Yes
	Zare, 2020 (28)	Rose	60	Pulse oximetry	Exp: 95.11 ± 3.35%; Control: 95.06 ± 2.69%	Exp: 95.91 ± 3.08% (2nd measurement); Control: 95.33 ± 2.65%	*p* = 0.001 (within-group at one time point)	No†
	Buyukbayram, 2021 (52)	Lavender	100	Pulse oximetry	Exp: 95.48 ± 2.66%; Control: 95.66 ± 2.72%	Exp: 95.14 ± 3.17%; Control: 94.40 ± 4.25%	*p* = 0.550 (Exp); *p* = 0.006 (Control ↓)	No
	Froutan, 2022 (26)	Rose + Lavender	62	Pulse oximetry	Exp: 96.68 ± 1.86%; Control: 95.63 ± 2.19%	Exp: 91.78 ± 3.55%; Control: 92.17 ± 2.28%	*p* < 0.001 (within-group both); MD = 0.55 (95% CI − 0.51 to 1.62); *p* = 0.301 (with inhalation); *p* = 0.099 (Group×measurement)	No†
	Silva, 2024 (33)	Lavender	52	Pulse oximetry	Exp: 94.65 ± 2.30%; Control: 94.46 ± 2.45%	Exp: 94.62 ± 2.32%; Control: 94.73 ± 2.10%	*p* = 0.974 (Exp); *p* = 0.613 (Control)	No
	Davari, 2021 (32)	Lavender	50	Pulse oximetry	Exp: \ ~ 93–97%; Control: \ ~ 93–97%	Exp: \ ~ 93–97%; Control: \ ~ 93–97%	*p* = 0.293	No
	Salamati, 2017 (24)	Lavender	40	Pulse oximetry	95.60%	94.99%	*p* = 0.5	No
	Ghasemi, 2017 (50)	Rose/Lavender	40	Pulse oximetry	\ ~ 96.5–99%	\ ~ 96.5–99%	*p* = 0.05	No
SaO_2_	Amini, 2022 (27)	Eucalyptus	70	Arterial blood gas analysis	Exp: 95.66 ± 2.91%; Control: 96.11 ± 1.99%	Exp: 97.2 ± 2.33%; Control: 95.14 ± 3.06% (Day 3)	*p* = 0.001	Yes
PaO_2_	Amini, 2022 (27)	Eucalyptus	70	Arterial blood gas analysis	Exp: 100.17 ± 42.28 mmHg; Control: 70.89 ± 36.2 mmHg	Exp: 103.03 ± 45.88 mmHg; Control: 82.72 ± 33.75 mmHg (Day 3)	*p* < 0.001	Yes
PIP	Amini, 2022 (27)	Eucalyptus	70	Ventilator monitoring	Exp: 19.63 ± 5.04 cmH₂O; Control: 16.60 ± 3.59 cmH₂O	Exp: 16.66 ± 4.55 cmH₂O; Control: 18.89 ± 4.15 cmH₂O (Day 3)	*p* = 0.001	Yes

Effect sizes represent the magnitude of change from baseline to post-intervention or the difference between intervention and control groups, as reported in individual studies. p-values and 95% confidence intervals are reported as published in the original studies; entries marked NS indicate the original study reported only “not significant” without an exact p-value. The frequent absence of 95% confidence intervals in the primary literature contributes to imprecision in the GRADE assessment. *p*-values are presented where available to indicate statistical significance (typically *p* < 0.05 considered significant). Oxygenation parameters measured via pulse oximetry (SpO_2_) provide non-invasive estimates, while arterial blood gas analysis (SaO_2_, PaO_2_) provides direct measurement of arterial oxygenation. Peak inspiratory pressure (PIP) was measured only in mechanically ventilated patients. The limited responsiveness of SpO_2_ to intervention in several studies may reflect a ceiling effect, as baseline values exceeding 94–95% leave minimal physiological capacity for detectable improvement due to the sigmoidal shape of the oxyhemoglobin dissociation curve.

Exp, experimental/intervention group; Control, control group; RR, respiratory rate; SpO_2_, peripheral oxygen saturation (measured via pulse oximetry); SaO_2_, arterial oxygen saturation (measured via arterial blood gas analysis); PaO_2_, partial pressure of arterial oxygen; PIP, peak inspiratory pressure; cmH₂O, centimeters of water; breaths/min, breaths per minute; mmHg, millimeters of mercury; N/A, not assessed; MD, mean difference (intervention − control); 95% CI, 95% confidence interval; NS, not significant (exact p-value not reported in original study); †Within-group effect only; no significant between-group interaction observed.

Nine studies examined the effect of aromatherapy on oxygenation parameters. The majority of studies (*n* = 8) measured peripheral oxygen saturation (SpO_2_) using pulse oximetry, while one study (27) measured both arterial oxygen tension (PaO_2_) and arterial oxygen saturation (SaO_2_) through arterial blood gas analysis. Overall, three studies (33.3%) reported statistically significant improvements in oxygenation parameters, while six studies (66.7%) found no significant between-group differences. The effects of aromatherapy on oxygenation parameters ranged from no significant change to improvements of approximately 1.5–2% in oxygen saturation. The most pronounced effects were observed in the Amini et al. study using nebulized Eucalyptus, which demonstrated significant improvements in both PaO_2_ and SaO_2_ in mechanically ventilated patients (27). The inconsistency in findings across studies may be attributed to several factors, including differences in the type of essential oil used (Eucalyptus vs. lavender), delivery method (nebulization vs. inhalation), patient populations, clinical settings, duration of intervention, and baseline oxygenation status. Aromatherapy interventions generally did not significantly affect SpO2 levels compared to control conditions in the majority of studies, suggesting that while certain specific interventions (particularly nebulized Eucalyptus) may improve oxygenation in mechanically ventilated patients, the effects of inhaled aromatherapy on oxygenation parameters remain limited in most clinical contexts. Only one study examined peak inspiratory pressure as an outcome measure, limiting the ability to draw generalizable conclusions. The Amini et al. study found that nebulized eucalyptus significantly reduced PIP in mechanically ventilated patients over a 3-day intervention period, with all recorded PIP values remaining within clinically acceptable ranges (16.6–19.6 cmH₂O) (27).

### Aromatherapy effects on non-cardiovascular and non-respiratory outcomes in the ICU

Beyond cardiovascular and respiratory parameters, several studies assessed psychological and clinical outcomes including anxiety, sleep quality, pain levels, relaxation, sedation indices, and mental stress (Table 5). These measures are presented to provide a complete picture of the evidence and to contextualize the physiological findings that are the primary focus of this review. Notably, the contrast between the certainty of evidence for these psychological outcomes and the physiological parameters represents a key finding: HIGH certainty for anxiety reduction contrasts with LOW to VERY LOW certainty for cardiovascular and respiratory parameters, reflecting the field’s historical emphasis on psychological measures.

**Table 5:** Effects of aromatherapy interventions on non-cardiovascular and non-respiratory outcome measures across included studies in critically ill and cardiac patients.

Outcome category	First author, year	Essential oil	N	Measurement tool	Baseline value (mean±SD)	Post-intervention value (mean±SD)	*P*-value	Significant effect
Anxiety (state)	Cho, 2013 (22)	Lavender + Chamomile + Neroli	56	STAI-KYZ, VAS	Exp: 5.5 ± 2.3; Control: 5.2 ± 2.7	Exp: 0.36 ± 0.73; Control: 3.11 ± 2.31	*p* < 0.001	Yes
	Mirbastegan, 2016 (49)	Lavender	60	Spielberger STAI (20–80)	Exp: 60.26 ± 9.29; Control: 56.60 ± 10.14	Exp: 41.56 ± 7.57; Control: 63.30 ± 5.19	*p* < 0.001	Yes
	Mahmoodabadipoor, 2024 (54)	*Citrus aurantium* + Chamomile	76	Spielberger STAI (20–80)	Exp: 57.9 ± 11.4; Control: 54.8 ± 9.3	Exp: 35.3 ± 8.4; Control: 50.1 ± 8.4	*p* < 0.001	Yes
	Pourshaikhian, 2024 (53)	Chamomile	154	STAI-6 (6–30)	Similar between groups (moderate–severe)	Exp: Mild (62.3%); Control: Moderate–Severe (98.7%)	*p* < 0.001	Yes
	Rajai, 2016 (29)	Lavender	60	DASS-21	Exp: 6.80 ± 3.84; Control: 7.23 ± 2.93	Exp: 6.63 ± 3.95; Control: 9.13 ± 4.55	*p* = 0.02	Yes
	Nategh, 2015 (31)	Lavender	110	HADS	Not specified	Significant reduction in intervention group	Significant	Yes
	Zare, 2020 (28)	Rose	60	Spielberger STAI (20–80)	Exp: 42.96 ± 10.69; Control: 44.13 ± 9.66	Exp: 42.66 ± 10.55; Control: 43.66 ± 10.78	*p* > 0.05	No
Anxiety (trait)	Mirbastegan, 2016 (49)	Lavender	60	Spielberger STAI (20–80)	Exp: 55.73 ± 10.22; Control: 54.13 ± 9.17	Exp: 44.53 ± 7.28; Control: 59.96 ± 7.17	*p* < 0.001	Yes
	Mahmoodabadipoor, 2024 (54)	*Citrus aurantium* + Chamomile	76	Spielberger STAI (20–80)	Exp: 50.0 ± 12.5; Control: 45.9 ± 10.8	Exp: 38.5 ± 8.2; Control: 44.8 ± 9.1	*p* = 0.002	Yes
Stress (perceived)	Cho, 2017 (25)	Lavender	60	Numeric Rating Scale (0–10)	Exp: 8.10 ± 1.61; Control: 6.27 ± 1.70	Exp: 3.73 ± 0.87; Control: 8.50 ± 0.90	*p* < 0.001	Yes
	Rajai, 2016 (29)	Lavender	60	DASS-21	Exp: 9.10 ± 4.25; Control: 7.90 ± 4.15	Exp: 8.63 ± 4.16; Control: 9.30 ± 4.59	*p* = 0.55	No
	Bikmoradi, 2015 (30)	Lavender	60	DASS-21	Day 2: \ ~ 15.1–15.3; Day 3: \ ~ 15.4	Day 2: 7.60 vs. 8.10; Day 3: 7.03 vs. 7.70	Day 2: *p* = 0.444; Day 3: *p* = 0.315	No
Stress (objective)	Cho, 2017 (25)	Lavender	60	HRV-based Index (1–10)	Exp: 7.73 ± 1.78; Control: 6.17 ± 3.11	Exp: 4.37 ± 1.97; Control: 8.00 ± 1.89	*p* < 0.001	Yes
Depression	Nategh, 2015 (31)	Lavender	110	HADS	Not specified	Significant reduction in intervention group	Significant	Yes
Sleep quality	Cho, 2013 (22)	Lavender + Chamomile + Neroli	56	VSH Sleep Scale	Exp: 53.0 ± 7.2; Control: 55.6 ± 15.4	Exp: 52.7 ± 13.8; Control: 36.2 ± 19.6	*p* = 0.001	Yes
	Cho, 2017 (25)	Lavender	60	Verran and Snyder-Halper Scale (0–80)	Exp: 65.13 ± 5.69; Control: 61.03 ± 7.83	Exp: 57.73 ± 5.61; Control: 25.80 ± 6.95	*p* < 0.001	Yes
	Davari, 2021 (32)	Lavender	50	St Mary’s Hospital Sleep Questionnaire (11–44)	Exp: 30.04 ± 4.82; Control: 30.36 ± 5.77	Exp: 25.08 ± 4.98; Control: 28.44 ± 6.62	*p* < 0.001	Yes
Pain	Silva, 2024 (33)	Lavender	52	Visual Numerical Scale (0–10)	Exp: 3.04 ± 3.26; Control: 2.77 ± 3.23	Exp: 1.35 ± 2.06; Control: 2.35 ± 3.27	*p* < 0.001	Yes
	Froutan, 2022 (26)	Damask Rose + Lavender	62	Visual Analog Scale (0–10)	Similar between groups	Exp: 4.5 ± 0.6; Control: 8.3 ± 1.4	*p* < 0.001	Yes
Agitation/sedation	Mashouf, 2017 (23)	Lavender	40	RASS	Approximately +2 (agitated)	Approximately 0 (calm and alert) at 180 min	*p* < 0.001	Yes
	Froutan, 2022 (26)	Damask Rose + Lavender	62	Bispectral Index (0–100)	Exp: 96.91 ± 1.73; Control: 97.20 ± 1.16	Exp: 71.43 ± 2.47; Control: 74.03 ± 1.13	*p* < 0.001	Yes
Sedative/analgesic consumption	Froutan, 2022 (26)	Damask Rose + Lavender	62	Ketamine (mg/kg)	Exp: 47.81 ± 5.52; Control: 49.33 ± 2.54	Exp: 0.00 ± 0.00; Control: 42.50 ± 10.40	*p* < 0.001	Yes
				Fentanyl (μg/kg)	Exp: 27.03 ± 7.61; Control: 50.0 ± 0.01	Exp: 0.00 ± 0.00; Control: 29.83 ± 22.76	*p* = 0.003	Yes
				Morphine (mg)	Exp: 4.03 ± 1.38; Control: 4.93 ± 0.37	Exp: 0.32 ± 1.22; Control: 4.13 ± 1.89	*p* < 0.001	Yes
				Propofol (mg/kg)	Exp: 18.59 ± 8.16; Control: 32.00 ± 8.87	Exp: 1.25 ± 4.92; Control: 53.00 ± 18.41	*p* < 0.001	Yes
				Midazolam (mg/kg)	Exp: 1.31 ± 0.52; Control: 2.65 ± 0.58	—	*p* < 0.001	Yes
Body temperature	Mashouf, 2017 (23)	Lavender	40	Monitoring device	37.2°C	36.9°C at 90 min	*p* < 0.001	Yes
	Silva, 2024 (33)	Lavender	52	Digital thermometer (°C)	Exp: 35.87 ± 0.58; Control: 36.13 ± 0.62	Exp: 35.90 ± 0.55; Control: 36.12 ± 0.60	*p* > 0.05	No
	Davari, 2021 (32)	Lavender	50	Digital thermometer (°C)	Exp: 36.79 ± 0.37; Control: 36.92 ± 0.42	Exp: 36.90 ± 0.28; Control: 36.92 ± 0.31	*p* > 0.05	No
	Bikmoradi, 2015 (30)	Lavender	60	Monitoring device	\ ~ 36.7–36.8°C	\ ~ 36.7–36.9 °C	NS	No
	Amini, 2022 (27)	Eucalyptus	70	Monitoring device	Exp: 37.18 ± 0.54; Control: 37.2 ± 0.59	Exp: 37.31 ± 0.74; Control: 37.21 ± 0.88 (Day 3)	*p* = 0.42	No
Sleepiness/relaxation	Silva, 2024 (33)	Lavender	52	Self-reported (Yes/No)	—	Exp: 61.54% sleepy; Control: 0%	*p* < 0.001	Yes
	Silva, 2024 (33)	Lavender	52	Researcher observation	—	Exp: 76.92% relaxed; Control: 7.69%	*p* < 0.001	Yes
Glasgow coma scale	Amini, 2022 (27)	Eucalyptus	70	Standard GCS	Exp: 6.23 ± 2.41; Control: 6.91 ± 2.8	Exp: 8.03 ± 3.63; Control: 7.71 ± 3.46 (Day 3)	*p* = 0.56	No
Central venous pressure	Salamati, 2017 (24)	Lavender	40	Barometer	10.20 mmHg	10.48 mmHg	*p* = 0.2	No

Effect sizes represent the magnitude of change from baseline to post-intervention or the difference between intervention and control groups, as reported in individual studies. P-values are presented where available to indicate statistical significance (typically p < 0.05 considered significant).

Exp, experimental/intervention group; Control, control group; STAI, State–Trait Anxiety Inventory; STAI-KYZ, Korean version of STAI; VAS, Visual Analog Scale; DASS-21, Depression Anxiety Stress Scales-21; HADS, Hospital Anxiety and Depression Scale; VSH, Verran and Snyder-Halpern; BIS, Bispectral Index; RASS, Richmond Agitation-Sedation Scale; GCS, Glasgow Coma Scale; CVP, Central Venous Pressure; HRV, Heart Rate Variability; NS, not significant; —, not measured or not reported.

#### Psychological outcomes

Anxiety was the most frequently assessed psychological outcome (7 studies), with 86% demonstrating significant reductions following aromatherapy. The most pronounced effects were reported by Cho et al. (22), with state anxiety scores decreasing from 5.5 ± 2.3 to 0.36 ± 0.73 (*p* < 0.001), and Mirbastegan et al. (26), with both state anxiety (60.26 ± 9.29 to 41.56 ± 7.57, *p* < 0.001) and trait anxiety (55.73 ± 10.22 to 44.53 ± 7.28, *p* < 0.001) significantly reduced using lavender aromatherapy. Notably, rose essential oil alone [Zare et al. (28)] showed no significant anxiolytic effect. Stress was assessed in four measures across three studies, with 50% demonstrating significant effects; Cho et al. (25) reported reductions in both perceived stress (*p* < 0.001) and objective stress index via heart rate variability (*p* < 0.001), while Rajai et al. (29) and Bikmoradi et al. (30) found no significant effects. Depression, assessed by Nategh et al. (31) using the HADS (Hospital Anxiety and Depression Scale), showed significant improvement (*p* < 0.05).

#### Sleep quality

All three studies (100%) evaluating sleep quality reported significant improvements with aromatherapy. Cho et al. (25) demonstrated maintained sleep quality in the intervention group versus significant decline in controls (57.73 ± 5.61 vs. 25.80 ± 6.95, *p* < 0.001), while Davari et al. (32) reported improved St Mary’s Hospital Sleep Questionnaire scores (30.04 ± 4.82 to 25.08 ± 4.98, *p* < 0.001).

#### Pain and sedation outcomes

Pain was assessed in two studies, both demonstrating significant reductions (100% success rate). Silva et al. (33) reported pain scores decreased from 3.04 ± 3.26 to 1.35 ± 2.06 (*p* < 0.001), while Froutan et al. (26) found significantly lower pain scores during burn dressing changes in the intervention group (4.5 ± 0.6 vs. 8.3 ± 1.4, *p* < 0.001). Sedation-related outcomes also showed 100% success rates; Mashouf et al. (23) demonstrated significant agitation reduction on the RASS (Richmond Agitation-Sedation Scale) (*p* < 0.001), and Froutan et al. (26) reported significant BIS reductions (96.91 ± 1.73 to 71.43 ± 2.47, *p* < 0.001). Notably, Froutan et al. (26) uniquely demonstrated significant reductions in all five sedative/analgesic medications assessed, including ketamine, fentanyl, morphine, propofol, and midazolam (all *p* < 0.01).

#### Other physiological parameters

Body temperature showed limited responsiveness, with only 1 of 5 studies (20%) demonstrating significant effects [Mashouf et al. (23): 37.2 °C to 36.9 °C, *p* < 0.001]. Central venous pressure [Salamati et al. (24), *p* = 0.2] and Glasgow Coma Scale [Amini et al. (27), *p* = 0.56] showed no significant changes. However, Silva et al. found that 76.92% of patients in the lavender group appeared facially relaxed compared to 7.69% in controls (*p* < 0.001) (33).

Overall, aromatherapy demonstrated highest efficacy for pain and sedation outcomes (100%), sleep quality (100%), and anxiety (86%), while other physiological parameters such as body temperature (20%), GCS (0%), and CVP (0%) showed minimal responsiveness. These findings suggest that aromatherapy exerts its most consistent effects on subjective and psychological outcomes rather than core physiological parameters.

##### Biochemical markers

Our comprehensive search specifically sought studies measuring biochemical markers such as cortisol, inflammatory cytokines (IL-6, TNF-*α*), catecholamines, or other stress biomarkers. None of the 18 studies included reported any biochemical outcomes.

### Subgroup analysis by essential oil type

Analysis of subgroups based on essential oil types showed significant differences in therapeutic effectiveness among the various oils (see Table 6). Lavender was the most extensively studied (11 studies, *n* = 710), demonstrating moderate cardiovascular success (64%), limited respiratory effects (27%), and strong efficacy for anxiety and sedation-related outcomes (71%). Chamomile achieved 100% success for both cardiovascular and anxiety outcomes, though this is based on a single study. Essential oil blends, particularly the lavender, roman chamomile, and neroli combination, showed superior results with 100% success rates for cardiovascular and psychological outcomes, suggesting potential synergistic effects of compound formulations. In contrast, rose as a single agent demonstrated limited efficacy across all outcome categories (cardiovascular: 17%; respiratory: 0%; pain/sedation: 0%), indicating it may be more effective in combination with other oils. Eucalyptus exhibited a distinct pharmacological profile with no cardiovascular effects but significant respiratory benefits (75%), including improved oxygenation and reduced peak inspiratory pressure, likely attributable to the bronchodilatory properties of 1,8-cineole (34). Notably, no studies measured biochemical markers such as cortisol or inflammatory mediators, representing a gap in understanding the physiological mechanisms underlying aromatherapy effects.

**Table 6:** Subgroup analysis of aromatherapy effects by essential oil type.

Essential oil	First author, year	N studies	N participants	Cardiovascular effects	Respiratory effects	Pain/sedation effects	Success rate by outcome
Lavender (*Lavandula angustifolia*)	Silva, 2024 (33); Buyukbayram, 2021 (52); Mirbastegan, 2016 (49); Davari, 2021 (32); Salamati, 2017 (24); Ghasemi, 2017 (50); Rajai, 2016 (29); Mashouf, 2017 (23); Ebrahimi Hosein Abadi, 2018 (51); Nategh, 2015 (31); Bikmoradi, 2015 (30)	11	710	Mixed results: Salamati: ↓SBP (*p* < 0.001), ↓DBP (*p* = 0.001), ↓HR (*p* = 0.03); Mashouf: ↓SBP, ↓DBP, ↓HR (all *p* < 0.001); Mirbastegan: ↓SBP (*p* = 0.042), ↓DBP (*p* = 0.048); Rajai: ↓HR (*p* = 0.02); Ebrahimi Hosein Abadi: ↓SBP day 2 (*p* = 0.046), ↓DBP day 1 (*p* = 0.029); Bikmoradi: ↓SBP/DBP at specific time points day 3; Silva: ↓MAP (*p* = 0.008); Buyukbayram, Davari, Ghasemi, Nategh: NS	Mixed results: Mashouf: ↓RR (*p* < 0.001), ↑SpO2 (*p* < 0.001); Silva: ↓RR (*p* = 0.011); Buyukbayram: ↓RR (*p* = 0.040); Ghasemi, Salamati, Davari, Bikmoradi: NS for RR; SpO2 generally NS	Positive: Silva: ↓Pain (*p* < 0.001); Mirbastegan: ↓Anxiety (*p* < 0.001); Mashouf: ↓Agitation (*p* < 0.001); Rajai: ↓Anxiety (*p* = 0.02); Davari: ↑Sleep quality (*p* < 0.001); Bikmoradi, Rajai: NS for stress	CV: 7/11 (64%); Resp: 3/11 (27%); Pain/Sedation: 5/7 (71%)
Chamomile (*Matricaria chamomilla*)	Pourshaikhian, 2024 (53)	1	154	Positive: ↓SBP (*p* < 0.05), ↓DBP (*p* < 0.001), ↓HR (*p* < 0.001)	Not measured	Positive: ↓Anxiety (*p* < 0.001)	CV: 3/3 (100%); Pain/Sedation: 1/1 (100%)
Lavender + Roman Chamomile + Neroli Blend (6:2:0.5)	Cho, 2013/2017 (22, 25)	1	56	Positive: ↓SBP (*F* = 6.77, *p* = 0.017), ↓DBP (*F* = 9.09, *p* = 0.004), ↓HR (*F* = 5.71, *p* < 0.001)	Not measured	Positive: ↓Anxiety (*p* < 0.001), ↓Stress (*p* < 0.001), ↑Sleep quality (*p* = 0.001)	CV: 3/3 (100%); Pain/Sedation: 3/3 (100%)
*Citrus aurantium* + Chamomile Blend	Mahmoodabadipoor, 2024 (54)	1	76	Partial: ↓HR (*p* = 0.039); NS for SBP	Not measured	Positive: ↓State anxiety (*p* < 0.001), ↓Trait anxiety (*p* < 0.001)	CV: 1/2 (50%); Pain/Sedation: 2/2 (100%)
Damask Rose + Lavender Blend	Froutan, 2022 (26)	1	62	Partial: ↓HR during inhalation (*p* < 0.001); NS for between-group BP differences	Mixed: ↓RR within-group (*p* = 0.001); NS for between-group interaction; SpO2: NS	Positive: ↓Pain (*p* < 0.001), ↓BIS (*p* < 0.001), ↓Sedative/analgesic doses (ketamine, fentanyl, morphine, propofol all significant)	CV: 0/1 (0% between-group); Resp: 0/1 (0% between-group); Pain/Sedation: 4/4 (100%)
Rose (Rosa damascena) Alone	Zare, 2020 (28); Ghasemi, 2017 (50)	2	100	Limited: Ghasemi: ↓SBP at 15, 45, 60 min post-extubation; NS for HR, DBP; Zare: Within-group SBP/DBP changes not clinically significant; NS for HR	NS: No significant RR changes; Zare: SpO2 ↑ at second measurement (*p* = 0.001) but not sustained	Zare: NS for anxiety	CV: 1/6 (17%); Resp: 0/4 (0%); Pain/Sedation: 0/1 (0%)
Eucalyptus (*Eucalyptus globulus*)	Amini, 2022 (27)	1	70	NS: No significant effects on HR (*p =* 0.82), SBP (*p =* 0.32), DBP (*p =* 0.42)	Positive: ↑PaO_2_ (*p* < 0.001), ↑SaO_2_ (*p* = 0.001), ↓PIP (*p* = 0.001); NS for RR (*p =* 0.88)	Not measured	CV: 0/3 (0%); Resp: 3/4 (75%); Biochem: 0/4 (0%)
GRAND Total	—	18	1,236	—	—	—	—

CV, cardiovascular; Resp., respiratory; NS, not significant; SBP, systolic blood pressure; DBP, diastolic blood pressure; HR, heart rate; RR, respiratory rate; SpO_2_, peripheral oxygen saturation; PaO_2_, partial pressure of arterial oxygen; SaO_2_, arterial oxygen saturation; PIP, peak inspiratory pressure; BIS, bispectral index; MAP, mean arterial pressure; N/A, not assessed; ↓significant decrease; ↑significant increase.

### GRADE assessment of certainty of evidence for aromatherapy in ICU patients

The certainty of evidence varied considerably across outcomes, ranging from very low to high certainty. The GRADE assessment revealed that aromatherapy interventions in ICU patients have the strongest evidence base for psychological outcomes, with more limited evidence for physiological parameters (Table 7). Downgrading occurred for the same reasons as systolic blood pressure. Evidence was downgraded for serious risk of bias and serious inconsistency due to mixed results across studies. Pain reduction was based on limited evidence from a single high-quality study showing significant pain reduction. Despite the study’s methodological rigor, evidence was downgraded for very serious imprecision due to the small sample size and lack of replication.

**Table 7:** GRADE assessment of certainty of evidence for aromatherapy outcomes in ICU patients.

Outcome	Effect_size	Effect_range	Risk_of_bias	Inconsistency	Indirectness	Imprecision	Certainty	Interpretation
Systolic blood pressure	Moderate decrease	−22.07 to −4.86 mmHg	Serious (−1)	Not serious (0)	Not serious (0)	Serious (−1)	⊕ ⊕ ○○ LOW	Aromatherapy may reduce SBP in ICU patients
Diastolic blood pressure	Moderate decrease	−14.4 to −3.2 mmHg	Serious (−1)	Not serious (0)	Not serious (0)	Serious (−1)	⊕ ⊕ ○○ LOW	Aromatherapy may reduce DBP in ICU patients
Heart rate	Moderate decrease	−20.23 to −6.89 bpm	Serious (−1)	Serious (−1)	Not serious (0)	Not serious (0)	⊕ ⊕ ○○ LOW	Aromatherapy may reduce heart rate in ICU patients
Respiratory rate	Small decrease	Variable effects	Serious (−1)	Serious (−1)	Not serious (0)	Serious (−1)	⊕○○○ VERY LOW	Very uncertain evidence for RR reduction
Oxygen saturation (SpO2)	Minimal increase	Mixed results	Serious (−1)	Very serious (−2)	Not serious (0)	Serious (−1)	⊕○○○ VERY LOW	Very uncertain evidence for SpO2 improvement
Pain	Large decrease	*p* < 0.001	Not serious (0)	Not assessed (−)	Not serious (0)	Very serious (−2)	⊕ ⊕ ○○ LOW	Limited evidence suggests pain reduction
Sleep quality	Large improvement	Effect size 1.28	Not serious (0)	Not serious (0)	Not serious (0)	Serious (−1)	⊕ ⊕ ⊕○ MODERATE	Aromatherapy probably improves sleep quality
Anxiety	Large decrease	Consistent reduction	Not serious (0)	Not serious (0)	Not serious (0)	Not serious (0)	⊕ ⊕ ⊕ ⊕ HIGH	Aromatherapy reduces anxiety in ICU patients

GRADE Certainty Ratings: ⊕ ⊕ ⊕ ⊕ HIGH: We are very confident that the true effect lies close to that of the estimate of the effect; ⊕ ⊕ ⊕○ MODERATE: We are moderately confident in the effect estimate; the true effect is likely to be close to the estimate of the effect, but there is a possibility that it is substantially different; ⊕ ⊕ ○○ LOW: Our confidence in the effect estimate is limited; the true effect may be substantially different from the estimate of the effect; ⊕○○○ VERY LOW: We have very little confidence in the effect estimate; the true effect is likely to be substantially different from the estimate of effect. Column Definitions: Effect_Size: Magnitude and direction of treatment effect; Effect_Range: Range of effects observed across studies or specific statistical measures; Risk_of_Bias: Assessment of study limitations (−1 serious, −2 very serious); Inconsistency: Assessment of heterogeneity between studies (−1 serious, −2 very serious); Indirectness: Assessment of applicability to target population and intervention (−1 serious, −2 very serious); Imprecision: Assessment of statistical precision based on sample size and confidence intervals (−1 serious, −2 very serious); Certainty: Final GRADE rating after considering all domains; Interpretation: Clinical interpretation of the evidence. Assessment methodology: Evidence from randomized controlled trials started as high certainty and was downgraded based on the five GRADE domains. Each domain could result in downgrading by one level (serious limitations) or two levels (very serious limitations). Non-randomized studies started as low certainty evidence.

Respiratory parameters demonstrated very low certainty evidence. Respiratory rate showed mixed results across 12 studies, with only four studies demonstrating significant decreases. Evidence was downgraded for serious risk of bias, serious inconsistency, and serious imprecision. Oxygen saturation (SpO_2_) had the weakest evidence base, with very inconsistent results across eight studies. Only one study showed a significant within-group increase, while three studies found no significant effects. Evidence was downgraded for serious risk of bias, very serious inconsistency, and serious imprecision.

The most common reasons for downgrading evidence certainty were:

1. Risk of bias (affecting 6 of 8 outcomes): Methodological limitations including lack of blinding due to the aromatic nature of interventions, small sample sizes, and single-center designs.
2. Imprecision (affecting 5 of 8 outcomes): Small individual study sample sizes and wide confidence intervals.
3. Inconsistency (affecting 2 of 8 outcomes): Heterogeneous results across studies, particularly for physiological parameters.

The evidence suggests that aromatherapy interventions are most reliably effective for psychological outcomes (anxiety, sleep) in ICU patients, with probable but less certain benefits for cardiovascular parameters, and very uncertain effects on respiratory parameters.

## Discussion

### Principal findings

This systematic review evaluated the effects of aromatherapy on physiological and biochemical outcomes in adult ICU patients. Eighteen studies comprising 1,236 participants were included, representing diverse ICU populations including post-cardiac surgery, mechanically ventilated, acute coronary syndrome, and burn patients. Our findings indicate that aromatherapy produces measurable effects on cardiovascular parameters with low certainty evidence, while evidence for respiratory effects remains very low certainty. A critical finding was the complete absence of biochemical marker assessment across all included studies, despite routine blood sampling availability in ICU settings—representing a major gap in mechanistic understanding.

### Cardiovascular effects

The effects of aromatherapy on cardiovascular parameters demonstrated considerable variability across included studies. For systolic blood pressure, statistically significant reductions were observed in 8 of 16 studies (50%), with effect size ranging from 5 to 22 mmHg. The most pronounced effects were observed in the Cho et al. and Mashouf et al. studies, which reported reductions of 16–22 mmHg (22, 23, 25). Even modest reductions in SBP (e.g., 5 mmHg) may be clinically relevant, as large-scale meta-analyses have demonstrated that a 5 mmHg reduction in SBP reduces the risk of major cardiovascular events by approximately 10% (35).

Diastolic blood pressure showed significant in 7 of 14 studies (50%) with effect size ranging from 3.7 to 14 mmHg. Notably, while systolic blood pressure has historically received greater attention in cardiovascular risk assessment, recent evidence from the 2023 European Society of Hypertension Guidelines suggests that diastolic blood pressure contributes independently to cardiovascular risk prediction, particularly when considered alongside systolic values in calculations such as mean arterial pressure (36).

Heart rate were observed in 7 of 16 studies (44%) with decreases up to 20 bpm in studies reporting significant effects. The Cho et al. (22, 25) and Mashouf et al. (23) studies again demonstrated the most pronounced effects. The inconsistency in findings across studies may be attributed to differences in essential oil type and concentration, duration and frequency of intervention, patient populations, clinical settings, and methodological approaches.

The notably larger effect sizes observed in the Cho et al. (22, 25) and Mashouf et al. (23) studies warrant consideration of potential explanatory factors. A 22 mmHg SBP reduction from a single inhalational session strains biological plausibility for inhaled essential oils acting via olfactory–limbic pathways, prompting consideration of statistical artifact alongside genuine intervention effects. Several characteristics may account for these findings: (1) Both studies enrolled patients with substantially elevated baseline hemodynamic values (HR ~ 92–97 bpm and SBP ~ 134–147 mmHg), and larger absolute treatment effects are structurally more probable when baselines are elevated, and regression to the mean (RTM)—the statistical tendency for extreme baseline values to move toward the population mean on repeat measurement, independently of any intervention—is a strong candidate alternative explanation, as well-documented in antihypertensive trial methodology; (2) Cho et al. employed a multi-component blend (lavender, Roman chamomile, and neroli at 6:2:0.5), and our subgroup analysis independently identified multi-component blends achieving 100% cardiovascular success versus 64% for lavender alone, suggesting potential synergistic interactions between linalool, apigenin, and geraniol; (3) Both studies enrolled patients during windows of maximal sympathetic activation—pre-procedural PCI and active agitation in mechanically ventilated patients—and interventions delivered during peak-stress windows may produce larger measurable effects. However, both studies had relatively small sample sizes, which may also inflate effect estimates through random variation.

### Respiratory effects

Aromatherapy demonstrated limited effects on respiratory parameters in most included studies. Respiratory rate was significntly reduced in only 3 of 12 studies (25%) with one additional study showing within-group changes without significant between-group differences. Oxygen saturation showed significant improvements in only 1 of 8 studies assessing SpO_2_ (12.5%).

However, eucalyptus oil exhibited a distinct pharmacological profile compared to other essential oils, demonstrating significant improvements in oxygenation parameters and respiratory mechanics in mechanically ventilated patients. Amini et al. reported that nebulized eucalyptus significantly improved PaO_2_, SaO_2_, tidal volume, and peak inspiratory pressure compared to controls (27). This respiratory efficacy is likely attributable to the bronchodilatory properties of 1,8-cineole (eucalyptol), the major constituent of eucalyptus oil (34). These findings suggest that essential oil selection should be tailored to therapeutic goals, with eucalyptus potentially preferred for respiratory optimization in mechanically ventilated patients.

### Non-cardiovascular/non-respiratory outcomes

Beyond cardiovascular and respiratory parameters, several studies assessed psychological and clinical outcomes including anxiety, sleep quality, pain, and sedation indices. These outcomes demonstrated higher success rates than physiological parameters: anxiety reduction (86% of studies), sleep quality improvement (100%), pain reduction (100%), and sedation outcomes (100%).

Froutan et al. demonstrated that mechanically ventilated burn patients receiving aromatherapy during wound dressing changes required significantly lower cumulative doses of all five co-administered sedative and analgesic agents—ketamine, fentanyl, morphine, propofol, and midazolam—with ketamine and fentanyl completely discontinued by the final measurement period (36). This sedative-sparing and analgesic-sparing effect may have important clinical implications for reducing medication-related adverse effects (e.g., respiratory depression, delirium, prolonged mechanical ventilation) in ICU settings.

### Clinical significance versus statistical significance

While the majority of studies demonstrated statistically significant effects, the clinical significance of these findings varies considerably across outcomes and populations (37, 38). The most clinically meaningful effects were observed in psychological parameters, where aromatherapy consistently produced substantial reductions in anxiety levels and improvements in sleep quality. These results align with evidence from a recent systematic review and meta-analysis of randomized controlled trials, which demonstrated that both inhalation and patch-based aromatherapy significantly lowered anxiety levels in hospitalized patients with acute myocardial infarction in the ICU. Further analysis indicated that the most pronounced effects were achieved with a two-day intervention and a total aromatherapy exposure time of 80 min (39). Additionally, a 2023 systematic review reported beneficial effects of aromatherapy for sleep quality and anxiety among ICU patients, albeit with very low but consistent evidence, highlighting the need for further robust research (40). These improvements are particularly relevant given that anxiety and sleep disturbances are common and distressing complications in ICU settings that can impact patient recovery and satisfaction.

For cardiovascular parameters, the clinical significance is more variable. Aromatherapy, using essential oils like lavender, has been shown to produce moderate reductions in systolic blood pressure and anxiety in patients with acute coronary syndrome or hospitalized cardiac patients (39, 41). These effects are most notably observed with inhalation therapy over short intervention periods in ICUs. A network meta-analysis published in 2025 reported that inhaled essential oil combinations, particularly those including lavender, *Matricaria recutita*, and neroli, significantly improved sleep quality among critically ill ICU patients, supporting complementary aromatherapy for this group (42). Several studies have noted that while statistical significance in blood pressure reduction is common, the clinical magnitude of these effects is moderate, and aromatherapy has greater impact on psychological than hemodynamic parameters in cardiac and respiratory ICU patients (41–43). In open heart surgery ICU settings, inhalation of lavender was associated with clinically meaningful reductions in blood pressure and heart rate, suggesting its utility for hemodynamic stability in perioperative care (24). Improvements in respiratory parameters, such as oxygenation and reduced peak inspiratory pressure, have been reported in some ICU patient groups, especially when aromatherapy is used alongside standard management for ventilator support, but more large-scale studies are needed (42). Comprehensive reviews highlight that aromatherapy is a non-invasive adjunct therapy, offering benefits in anxiety reduction, modest cardiovascular improvement, and enhanced sleep without significant adverse effects, though methodological rigor remains an important need for future research (6, 44).

### Evidence certainty and outcome prioritization

A key finding of this review is the disparity between evidence certainty for psychological versus physiological outcomes. High certainty evidence for anxiety reduction and moderate certainty evidence for sleep improvement contrast sharply with low and very low certainty evidence for cardiovascular and respiratory parameters, respectively. This pattern reflects the field’s historical emphasis on psychological measures rather than a true difference in aromatherapy’s effectiveness across outcome domains.

The included studies varied considerably in their outcome prioritization, with many designating psychological measures as primary endpoints while treating physiological parameters as secondary outcomes. While the ICU provides continuous physiological monitoring capability that should theoretically facilitate high-quality hemodynamic data, existing trials failed to control for the dominant pharmacological and mechanical determinants of cardiovascular parameters in critically ill patients, undermining causal inference for physiological outcomes even when measurement precision was high. This disparity directly tests and refutes the hypothesis motivating our review: that the ICU’s controlled monitoring environment would generate high-quality physiological evidence.

### Essential oil subgroup analysis

Subgroup analysis by essential oil type revealed differential efficacy patterns. Multi-component blends (lavender combined with chamomile, neroli, or rose) achieved 100% success rates for cardiovascular outcomes, compared to 64% for lavender monotherapy. This finding suggests potential synergistic interactions between essential oil constituents that may enhance therapeutic effects.

Lavender (*Lavandula angustifolia*), used in 13 of 18 studies either alone or in combination, demonstrated the most consistent evidence base. Its primary bioactive constituents—linalool and linalyl acetate—have demonstrated anxiolytic and sedative properties through modulation of GABAergic neurotransmission in preclinical studies. However, the predominance of lavender in the literature limits conclusions about other potentially efficacious essential oils.

Chamomile, used in three studies, demonstrated consistent cardiovascular and anxiolytic effects. Rose oil, used in three studies, showed variable cardiovascular effects but consistent anxiolytic properties. Eucalyptus, evaluated in only one study, demonstrated unique respiratory benefits not observed with other oils, suggesting mechanism-specific applications.

### Practical clinical implications

The evidence suggests several practical applications for aromatherapy in ICU settings. Lavender-based interventions emerge as the most evidence-supported option, with consistent effects across multiple outcomes and populations (45, 46). The interventions are generally described as “simple, safe, and inexpensive”, making them accessible for routine clinical implementation (42, 46). The interventions are characterized as fast-acting and easy to apply, attributes that are advantageous in the demanding ICU environment (45, 46). Specific clinical applications include: pre-procedural anxiety reduction in cardiac patients (45, 46), post-operative pain and sleep management (26, 47), agitation control in mechanically ventilated patients (23, 27), and hemodynamic stabilization in cardiac care units (24). The potential for reducing sedative and analgesic requirements suggests aromatherapy could serve as an adjunct to conventional pain management protocols (46). Implementation considerations include the need for standardized protocols, staff training, and consideration of patient preferences and potential contraindications. The variety of delivery methods and dosing regimens across studies suggests the need for institutional protocols to ensure consistent application.

### Limitations

Several methodological limitations affect the interpretation and generalizability of these findings. This systematic review was not prospectively registered in PROSPERO or another recognized registry. While a protocol was developed before conducting the review, the lack of formal registration reduces transparency regarding pre-specified methods and increases the potential for post-hoc analytical decisions. Our PubMed search identified 9 of 18 included studies (50.0%). This limited retrieval likely reflects our search string construction (which omitted population and outcome filters) and the absence of key databases (CINAHL, EMBASE, Scopus) rather than inherent limitations of lexical database searching. Future systematic reviews in this field should employ comprehensive multi-database strategies with fully specified PICO elements.

The substantial heterogeneity in study designs, essential oil types, administration methods, and outcome measures reflects both the current state of the evidence base and our deliberately broad inclusion criteria. We adopted permissive eligibility criteria—accepting any adult ICU subpopulation, any essential oil type, and any administration route—to provide comprehensive field coverage. However, this methodological decision directly contributed to the heterogeneity that precluded meta-analysis. We acknowledge this heterogeneity is partially a consequence of our methodological choices rather than solely attributable to the literature.

The predominance of cardiovascular ICU populations (13/18 studies) limits generalizability to other ICU contexts. Additionally, the included studies did not consistently report validated severity scores such as the Acute Physiology and Chronic Health Evaluation II (APACHE II) or Sequential Organ Failure Assessment (SOFA), precluding subgroup analysis by illness severity. The included studies did not adequately control for ICU-specific confounders including vasoactive medications, sedation protocols, and mechanical ventilation parameters, creating bidirectional risks of effect inflation (when concurrent medication weaning or sedation deepening coincides with aromatherapy administration) and effect masking (when ongoing vasopressor titration overrides aromatherapy-mediated changes), and limiting causal inference for cardiovascular outcomes. Most included studies demonstrated some concerns or high risk of bias, particularly in blinding domains. The inherent difficulty in blinding aromatherapy interventions due to distinctive odors poses methodological challenges that may inflate effect estimates. Only three included studies (24, 36, 39) reported 95% confidence intervals for between-group differences. This deficiency in the primary literature directly contributes to the imprecision domain of our GRADE assessment. The predominance of positive findings across included studies raises concern for publication bias, whereby studies demonstrating null or negative results may be underrepresented in the published literature.

## Conclusion

This systematic review demonstrates that aromatherapy interventions produce measurable effects in adult ICU patients. Low certainty evidence suggests potential cardiovascular benefits, including blood pressure and heart rate reductions, though these findings require cautious interpretation given inadequate control for ICU-specific confounders including vasoactive medications and sedation. Evidence for respiratory effects remains insufficient to support clinical recommendations.

The complete absence of biochemical marker assessment across all 18 studies represents a critical evidence gap that limits mechanistic understanding. The disparity between high certainty evidence for anxiety reduction and low/very low certainty evidence for cardiovascular and respiratory parameters highlights the field’s historical emphasis on psychological measures while objective physiological and biochemical mechanisms remain inadequately investigated. This gap likely reflects several converging factors: aromatherapy research has historically been conducted within nursing and complementary medicine paradigms that prioritize patient-reported outcomes over biochemical endpoints (48); the cost and infrastructure for cortisol, catecholamine, and inflammatory cytokine assays exceed many trial budgets; ethical review boards may resist additional research-only blood draws in critically ill patients; and disciplinary segregation between aromatherapy and clinical pharmacology investigators has limited methodological cross-fertilization. As a consequence, the observed hemodynamic changes cannot be mechanistically attributed to genuine neuroendocrine modulation, peripheral pharmacological effects, placebo response, or statistical artifact (8).

Future research should prioritize rigorous trial designs with prospective registration, biochemical marker assessment, standardized intervention protocols, and adequate control for vasoactive medications and sedation to establish physiological mechanisms and clinical significance. Aromatherapy represents a safe, low-cost adjunctive intervention that can be integrated into holistic ICU care, with current evidence most strongly supporting its use for anxiety management and sleep promotion.

## Data Availability

Publicly available datasets were analyzed in this study. This data can be found at: no new data were created or analyzed in this study. Data sharing is not applicable to this article as it relies on data extracted from previously published studies.
